# Prospective Single-Site Experience with Radiofrequency-Targeted Vertebral Augmentation for Osteoporotic Vertebral Compression Fracture

**DOI:** 10.1155/2013/791397

**Published:** 2013-10-20

**Authors:** Franklin G. Moser, Marcel M. Maya, Laura Blaszkiewicz, Andrea Scicli, Larry E. Miller, Jon E. Block

**Affiliations:** ^1^Cedars-Sinai Medical Center, 8700 Beverly Boulevard, Los Angeles, CA 90048, USA; ^2^DFINE, Inc., 3047 Orchard Parkway, San Jose, CA 95134, USA; ^3^Miller Scientific Consulting, Inc., 26 Portobello Road, Arden, NC 28704, USA; ^4^The Jon Block Group, 2210 Jackson Street, Suite 401, San Francisco, CA 94115, USA

## Abstract

Vertebral augmentation procedures are widely used to treat osteoporotic vertebral compression fractures (VCFs). We report our initial experience with radiofrequency-targeted vertebral augmentation (RF-TVA) in 20 patients aged 50 to 90 years with single-level, symptomatic osteoporotic VCF between T10 and L5, back pain severity > 4 on a 0 to 10 scale, Oswestry Disability Index ≥ 21%, 20% to 90% vertebral height loss compared to adjacent vertebral body, and fracture age < 6 months. After treatment, patients were followed through hospital discharge and returned for visits after 1 week, 1 month, and 3 months. Back pain severity improved 66% (*P* < 0.001), from 7.9 (95% CI: 7.1 to 8.6) at pretreatment to 2.7 (95% CI: 1.5 to 4.0) at 3 months. Back function improved 46% (*P* < 0.001), from 74 (95% CI: 69% to 79%) at pretreatment to 40 (95% CI: 33% to 47%) at 3 months. The percentage of patients regularly consuming pain medication was 70% at pretreatment and only 21% at 3 months. No adverse events related to the device or procedure were reported. RF-TVA reduces back pain severity, improves back function, and reduces pain medication requirements with no observed complications in patients with osteoporotic VCF.

## 1. Introduction

Osteoporosis is a devastating disease characterized by low bone density, microarchitectural deterioration of bone tissue, and increased susceptibility to fracture. Vertebral compression fracture (VCF) is a common manifestation of osteoporosis in the elderly with an incidence of 700,000 cases per year in the United States alone [1]. VCFs can precipitate in a downward spiral of physical functioning characterized by chronic back pain, limited mobility, functional impairment, and kyphosis resulting in reduced pulmonary capacity, loss of stature, and greater risk of subsequent nonvertebral and additional vertebral fractures [2, 3]. Vertebral compression fractures also represent a significant economic burden in the United States, accounting for an estimated 17.5 billion dollars in annual medical costs [4]. As the population continues to age, VCFs will remain a serious public health concern.

Conservative treatment of painful VCFs, such as bed rest, braces, and/or narcotic analgesic medication, has only modest short-term effectiveness and is associated with poor long-term outcomes, including exacerbation of bone loss, increased risk of subsequent fracture, decreased mobility, and increased mortality [5–7]. Percutaneous techniques such as vertebroplasty and balloon kyphoplasty (BK) are minimally invasive treatments for acutely painful VCFs and have been used with increasing frequency over the last two decades [8]. Vertebroplasty involves injecting polymethylmethacrylate (PMMA) into the collapsed vertebrae to reduce pain via vertebral stabilization. Balloon kyphoplasty involves creation of a cavity before cement injection, which theoretically provides an advantage by correcting for sagittal alignment and reducing the risk of cement leakage. Vertebroplasty and BK are both safe and effective procedures for treatment of osteoporotic VCF although BK may result in better clinical outcomes and fracture reduction compared to vertebroplasty in patients with significant vertebral body height loss [9].

Radiofrequency-targeted vertebral augmentation (RF-TVA) is a novel percutaneous vertebral augmentation technique that was developed to address some of the limitations of BK while maintaining the benefit of vertebroplasty and BK. The purpose of this study was to assess the initial safety and effectiveness of RF-TVA in osteoporotic patients with symptomatic VCF.

## 2. Methods

### 2.1. Ethics

All study procedures were conducted in accordance with the ethical principles stated in the Declaration of Helsinki, and this research was approved by the Institutional Review Board at the investigative site. All patients signed an informed consent before participation in any study activities. This study was registered at ClinicalTrials.gov (NCT01839682).

### 2.2. Study Design

This prospective, single-site postmarket study was conducted to evaluate the safety and effectiveness of RF-TVA in patients with single-level, symptomatic osteoporotic VCF.

### 2.3. Patients

Twenty-three consecutive patients were enrolled in this study. Main inclusion criteria included age between 50 and 90 years, single-level osteoporotic VCF located between T10 and L5, back pain severity > 4 on a 0-to-10 scale, Oswestry Disability Index (ODI) ≥ 21%, vertebral height loss between 20% and 90% compared to adjacent vertebral body, and fracture age < 6 months. Main exclusion criteria included primary tumor or metastasis, neurologic deficit associated with the index level, pedicle fracture, significant kyphosis (>30°) or translation (>4 mm), and active infection.

### 2.4. Pretreatment Evaluations

Patients underwent a physical examination, complete medical history, and radiographic imaging studies including thoracolumbar lateral and anteroposterior radiographs to confirm the presence and characteristics of the VCF. Symptomatic levels were confirmed with radionuclide bone scan and computed tomography or magnetic resonance imaging. Patient-reported back pain severity was quantified using an 11-point (0 to 10) numeric scale. Back-specific functional disability was self-reported with the ODI (version 2) on a 0 to 100% scale [10]. 

### 2.5. Procedural Details

**Figure 1:**
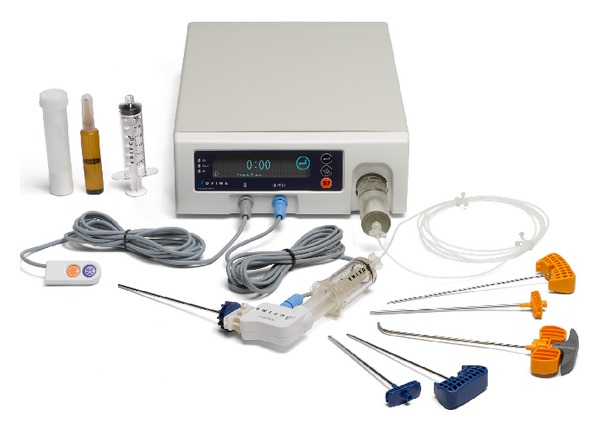
StabiliT Vertebral Augmentation System (DFINE, Inc., San Jose, CA, USA).

**Figure 2:**
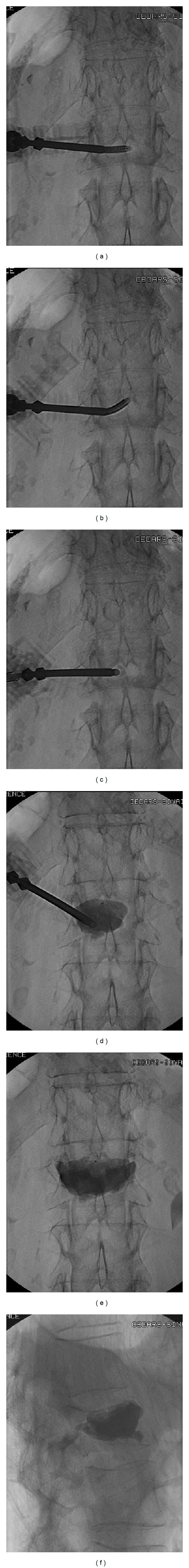
Stepwise unipedicular RF-TVA procedure including (a) controlled intravertebral cavity creation with MidLine Osteotome articulated across vertebral midline, (b) osteotome reinserted cranially to create additional site-specific cavities, (c, d) targeted delivery of StabiliT ER2 ultrahigh viscosity PMMA, and (e) anteroposterior and (f) lateral views demonstrating extensive PMMA trabecular interdigitation and controlled vertebral height restoration.

All procedures were performed by an experienced interventional neuroradiologist in a biplane angiographic suite. Patients were first placed in the prone position and prepped and draped in the usual manner. RF-TVA was performed via a unipedicular approach with site-specific cavity creation followed by remotely controlled delivery of ultrahigh viscosity PMMA (StabiliT ER^2^ Bone Cement) using the StabiliT Vertebral Augmentation System (DFINE, Inc, San Jose, CA, USA) (Figure 1). After placement of a working cannula, an initial cavity was created by inserting a straight coring osteotome (VertecoR StraightLine Osteotome) that permits biopsy. A navigational osteotome (VertecoR MidLine Osteotome) was then inserted through the working cannula. The MidLine Osteotome was navigated through the vertebral body by rotating the handle to articulate the distal beveled tip as the osteotome was advanced through the vertebral body to the anterior third and across the midline in a controlled fashion (Figure 2(a)). The MidLine Osteotome was then reoriented, and additional passes were made to create site-specific cavities. This resulted in well-defined cavities with minimal disruption of adjacent trabeculae that served as preferential paths for cement delivery (Figures 2(b) and 2(c)). The PMMA, which has an extended working time compared to standard PMMA, was then converted to ultrahigh viscosity PMMA as it passed through the activation element (where it was heated by application of radiofrequency) immediately prior to entering the delivery cannula, thus providing a constant viscosity throughout the procedure. The ultrahigh viscosity PMMA was delivered at a continuous rate of 1.3 cc/min using a remotely controlled automated hydraulic delivery system. Intermittently during PMMA delivery, intravertebral filling was fluoroscopically monitored and terminated when the treating physician deemed that there was a risk of extravasation or that the fill was adequate (Figure 2(d)). In cases where cement delivery was halted due to risk of extravasation but prior to optimal filling, the long working time allowed for cement delivery to be reinitiated after cement in the areas of potential extravasation had set. The remote control cement delivery feature of this device allows the physician user to perform the procedure at a safe distance from the fluoroscopic unit (>10 feet), providing a marked reduction in operator radiation exposure. Final imaging was then obtained to confirm alignment and fill (Figures 2(e) and 2(f)).

### 2.6. Outcomes

Back pain severity was assessed at discharge and at 1 week, 1 month, and 3 months posttreatment. ODI was measured at 1 week, 1 month, and 3 months posttreatment. Use of pain medications was reported according to the World Health Organization three-step analgesic pain ladder, a semiquantitative scale used to describe use of pain management drugs [11]. Current pain medication use is defined as 0 for no current pain medication use, 1 for current use of nonopioid pain medication (e.g., paracetamol), 2 for current use of weak opioid pain medication (e.g., codeine), and 3 for current use of strong opioid pain medication (e.g., morphine). Adverse events were collected throughout the study and were defined as any reported complication, regardless of the relationship with the procedure or device.

### 2.7. Data Analysis

Data were analyzed using Predictive Analytics Software (v. 18, SPSS, Inc., Chicago, IL, USA). All continuous data were verified to be normally distributed using the Shapiro-Wilk test. Continuous data were reported as mean ± 95% confidence interval (CI) using exact methods, and categorical data were reported as frequencies and percentages. Longitudinal changes in back pain severity and ODI values were assessed with repeated measures analysis of variance. Clinical success was defined as an improvement of ≥30% compared to pretreatment values for back pain severity and ODI, respectively [12, 13]. Pain medication usage during the study was assessed with the Wilcoxon signed rank test.

## 3. Results

### 3.1. Patient Characteristics

Twenty-three patients were enrolled in this study. Mean patient age was 79 (95% CI: 74 to 83) years. The most common type of VCF was a wedge fracture. Mean vertebral body collapse was 27% (95% CI: 21% to 33%). Patients had severe back pain (7.9, 95% CI: 7.1 to 8.6) and dysfunction (ODI: 74%, 95% CI: 69% to 79%) at pretreatment. Most patients also presented with associated diseases, most commonly hypertension (*n* = 14), coronary artery diseases (*n* = 8), and diabetes mellitus (*n* = 5) (Table 1).

### 3.2. Treatment Details

Three patients were unable to receive treatment due to difficulty with placing a cannula in patients with a high body mass index. Ultimately, 20 patients underwent treatment with the RF-TVA system between June 2009 and February 2011. The procedures were performed with deep sedation and local anesthetic in 19 of 20 cases. Mean procedure time was 19 (95% CI: 14 to 23) minutes with average cement volume delivery of 4.0 (95% CI: 3.3 to 4.7) cc. No procedural complications or cement extravasation was identified. Hospital stay following the RF-TVA procedure was minimal, with same day discharge in 70% of patients and next day discharge in 30% of patients.

### 3.3. Patient-Reported Outcomes

**Figure 3:**
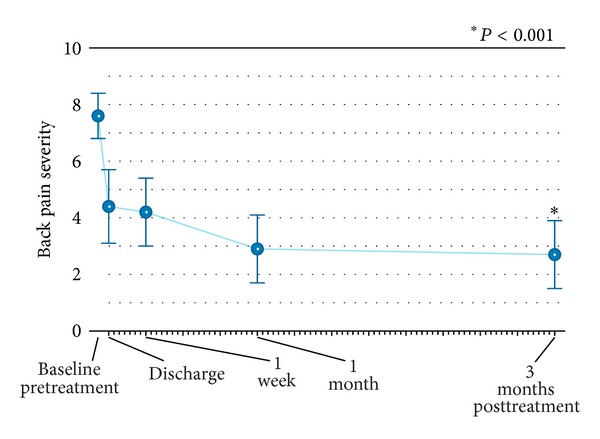
Improvement in back pain through 3 months following radiofrequency-targeted vertebral augmentation. Values are mean ± 95% confidence intervals.

**Figure 4:**
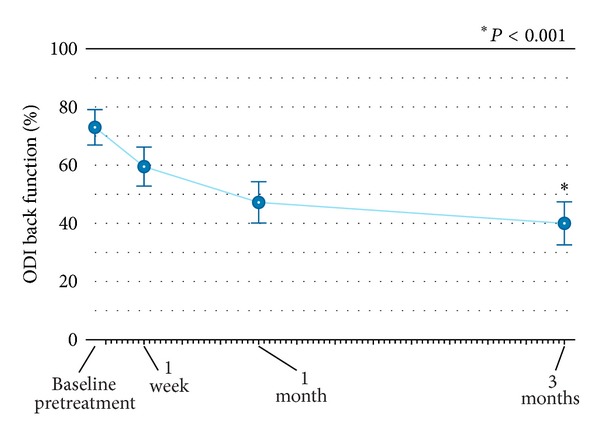
Improvement in back function through 3 months following radiofrequency-targeted vertebral augmentation. Values are mean ± 95% confidence intervals. ODI: Oswestry Disability Index.

Back pain severity rapidly improved from 7.9 (95%: 7.1 to 8.6) at pretreatment to 4.4 (95% CI: 3.1 to 5.6) at hospital discharge. Over 3-month follow-up, back pain continued to improve with mean scores of 2.7 (95% CI: 1.5 to 4.0) at 3 months, representing a 66% (*P* < 0.001) overall improvement (Figure 3). Back function similarly improved from 74% (95% CI: 69% to 79%) at pretreatment to 60% (95% CI: 54% to 66%) at 1 week and 40% (95% CI: 33% to 47%) at 3 months, representing a 46% (*P* < 0.001) overall improvement (Figure 4). Clinical success rates were 84% for back pain severity and 79% for ODI at 3-month follow-up.

### 3.4. Pain Medication Consumption

Patients were able to wean off pain medications over the 3-month follow-up period. At pretreatment, 70% of patients were routinely consuming pain medications, including 60% who regularly consumed opioids. At 3 months, only 21% of patients were regularly consuming pain medication with 16% consuming opioids (Table 2).

### 3.5. Adverse Events

No adverse events related to the device or procedure were reported during the study (0 of 20 patients). One patient died from causes unrelated to the procedure before the 3-month follow-up visit.

## 4. Discussion

The RF-TVA system is a novel, safe technique for percutaneous vertebral augmentation in osteoporotic patients with symptomatic VCFs. Our early experience with this device demonstrates significant improvements in back pain severity and back function and reduced pain medication use with no observed complications.

The results of this study are consistent with outcomes observed in an *ex vivo *biomechanical study of the RF-TVA system [14]. This study reported that RF-TVA produced more discrete cavities and less native trabecular destruction compared to marked trabecular destruction observed with BK. Additionally, RF-TVA consistently showed a well-identified focal area of PMMA with an extensive peripheral zone of PMMA interdigitation, providing mechanical interlock into the adjacent intact trabecular matrix. Additionally, several retrospective studies of RF-TVA for treatment of VCFs have demonstrated consistent improvements in back pain severity and ODI and no procedural complications [15–17]. The results of these studies and of the current prospective study provide increasing evidence that treatment with RF-TVA is optimized by the targeted delivery and interdigitation of ultrahigh viscosity RF-heated cement into the remaining trabeculae. 

In vertebral augmentation procedures, PMMA cement may leak laterally to the soft tissues, superiorly or inferiorly into the adjacent disc space, or posteriorly, where it may involve the exiting nerve root or the spinal canal [18]. The RF-TVA system utilizes a fill control mechanism to ensure a controlled, constant rate of bone cement delivery, which encourages PMMA interdigitation and may enhance fracture stability. Additionally, the Midline Osteotome allows unipedicular access to the vertebra, enables targeted site- and size-specific cavity creation across the vertebral midline, and spares cancellous bone, thereby creating preferential pathways for the high-viscosity bone cement. Our first experience in 20 patients performing the RF-TVA procedure yielded no cement extravasation. These results compare favorably to cement leakage rates of 7% to 72% reported with kyphoplasty and vertebroplasty [19–23]. The ultrahigh viscosity PMMA also allows for an extended working time, which is particularly advantageous in patients with multiple levels requiring treatment or in vertebrae at risk of extravasation. The ability to discontinue cement delivery for extended periods of time (due to the long working time cement and RF activation immediately prior to delivery), while that cement in the patient hardens serving as a barrier, enables more complete augmentation with decreased potential for extravasation.

Minimizing physician exposure to radiation during minimally invasive procedures for treatment of VCFs remains a concern. Although the use of methods such as shielding and intermittent fluoroscopy has dramatically reduced physician users exposure to radiation during traditional vertebral augmentation procedures [24, 25], whole body radiation exposure rates of 0.3–1.1 microsieverts per minute during device positioning and 0.2–2.9 microsieverts during cement injection have been reported [26]. For reference, a mammogram exposes a patient to a whole body radiation dose of 0.4 microsieverts. The use of a hand switch to remotely control cement delivery in StabiliT System allows the operator to remain as far as 20 feet from the radiation source versus 0 to 2 feet for competitive devices [27, 28], thereby exponentially reducing user radiation exposure. This is a significant advantage to physicians who perform a high volume of vertebral augmentation cases.

Strengths of this study included the prospective design, use of validated measures of patient-reported outcomes, and strict inclusion/exclusion criteria. Limitations of this study include lack of a control group, inclusion of only single-level fracture cases, and a relatively short follow-up period.

## 5. Conclusions

The results from our initial clinical experience showed that RF-TVA dramatically reduces back pain severity, improves back function, and reduces pain medication requirements with no observed complications in patients with osteoporotic VCF. Remotely controlled cement delivery may reduce physician radiation exposure during vertebral augmentation procedures. Overall, the initial clinical results of this prospective trial are promising and warrant further study in larger series with longer follow-up periods.

## Figures and Tables

**Table 1:** Patient baseline characteristics.

Characteristic	Value
	(*n* = 23)
Age, mean ± SD, y	79 ± 10
Female, *n* (%)	**17** (74)
Body mass index, mean ± SD, kg/m^2^	27 ± 7
Back pain severity, mean ± SD, cm	7.9 ± 1.8
Oswestry Disability Index (ODI), mean ± SD, %	74 ± 12
Vertebral collapse, mean ± SD, %	27 ± 14
Fracture age, mean ± SD, days	13 ± 13
Fracture type, *n* (%)	
Wedge	**14** (61)
Crush	**7** (30)
Concave	**2** (9)
Medical history*, *n* (%)	
Hypertension	**14** (61)
Coronary artery disease	**8** (35)
Diabetes mellitus	**5** (22)
Previous spine surgery	**4** (17)
COPD	**3** (13)

*Reported on variables with frequency > 10%, sum of percentages > 100% due to multiple conditions per patient.

**Table 2:** Pain medication use before and 3 months after radiofrequency-targeted vertebral augmentation.

WHO drug category	Pretreatment	3 months	*P* value
	(*n* = 20)	(*n* = 19)	
Class 0, no drugs	**6** (30)	**15** (79)	<0.01
Class 1, nonopioid analgesics	**2** (10)	**1** (5)	
Class 2, weak opiate derivative	**10** (50)	**3** (16)	
Class 3, strong opiate derivative	**2** (10)	**0** (0)	

*P* value compares the ordinal distribution of pain medication use from pretreatment to 3 months; calculation based on paired cases (*n* = 19).
